# A Structured Approach to Discharge Documentation: Lessons from Dongola Specialized Hospital

**DOI:** 10.7759/cureus.81696

**Published:** 2025-04-04

**Authors:** Abubakr Muhammed, Fakher Aldeen Raft Fakher Aldeen Noman, Mohaned Altijani Abdalgadir Hamdnaalla, Waddah Ahmed, Ahmed Mohamed, Mawada Taha, Ahmed Akasha, Mohammed Ali Mohammed Ali, Samir Ibrahim, Mohammed Osman, Mohey Aldien Ahmed Elamin Elnour, Malaz Alamin, Rayan Elmutaz Mohamed Mahmoud, Lubaba A Alkhider, Mohamed Ziada, Mawada M Ali Mohamed, Esraa Elmutaz Mohamed Mahmoud, Mozaffar Sirelkhatim Elzain Abdalla, Mohammad Alrawi, Mohammad Alzain Adam

**Affiliations:** 1 Surgery, University of Gezira, Wad Madani, SDN; 2 Internal Medicine, Dongola Specialized Hospital, Dongola, SDN; 3 Orthopedics and Trauma, Gezira Center for Orthopedic Surgery and Traumatology, Wad Madani, SDN; 4 General Surgery, Dongola Specialized Hospital, Dongola, SDN; 5 Neurological Surgery, Dongola Specialized Hospital, Dongola, SDN; 6 Anaesthesia, Dongola Specialized Hospital, Dongola, SDN; 7 Emergency, Dongola Specialized Hospital, Dongola, SDN

**Keywords:** care continuity, discharge documentation, patient safety, resource-limited settings, standardized discharge card

## Abstract

Background

Effective discharge documentation is essential for ensuring patient safety, care continuity, and communication among healthcare providers. However, in resource-limited settings like Sudan, documentation quality is often suboptimal, leading to gaps in care and poor patient outcomes. This quality improvement project (QIP) at Dongola Specialized Hospital aimed to address these challenges by implementing a standardized discharge card and providing targeted staff training.

Methods

The study was conducted over two cycles, with data collected from 50 discharge cards in each cycle, selected using a simple randomization technique. The first cycle assessed baseline documentation practices, revealing significant inconsistencies. A standardized discharge card was then developed and implemented, accompanied by training sessions for healthcare providers. The second cycle evaluated the intervention’s effectiveness, measuring compliance and completeness of patient information (e.g., clinical summaries, discharge plans, and medication lists). Feedback from healthcare providers and patients was also collected to assess the new system’s impact.

Results

The intervention led to significant improvements in discharge documentation quality. Compliance with the new format increased from 66% in the first cycle to 92% in the second cycle. Completeness of patient information reached 100%, while clinical summaries and discharge plans improved by 40% and 30%, respectively. Medication list accuracy also increased to 88%. Preliminary data indicated a 15% reduction in readmission rates, attributed to clearer postdischarge instructions. However, challenges such as incomplete documentation in certain sections and time constraints for healthcare providers remained.

Conclusion

The implementation of a standardized discharge card significantly improved the quality of discharge documentation at Dongola Specialized Hospital, contributing to better patient outcomes and reduced readmission rates. The findings highlight the importance of structured documentation and regular audits in enhancing patient safety and care continuity, particularly in resource-limited settings. Ongoing efforts are needed to address remaining challenges, such as incomplete documentation and time constraints, to ensure sustained improvements in the discharge process. This study serves as a model for similar healthcare facilities aiming to improve documentation practices and patient care.

## Introduction

Effective medical documentation is a cornerstone of high-quality patient care, ensuring continuity, safety, and efficient communication among healthcare providers. Discharge summaries, in particular, play a critical role in transitioning patient care from hospital settings to primary care or other healthcare facilities. They provide essential information about the patient’s diagnosis, treatment, medications, and follow-up plans, which are vital for ongoing management. However, studies have shown that incomplete or inadequate discharge documentation can lead to gaps in care, medication errors, and poor patient outcomes [1]. In resource-limited settings, such as Sudan, where healthcare systems face significant challenges, the quality of documentation is often suboptimal, further exacerbating these issues [2].

The importance of structured and standardized discharge documentation has been emphasized in various studies. For instance, an audit conducted in a tertiary care hospital revealed that the introduction of a discharge summary template significantly improved the completeness and quality of documentation, particularly in areas such as medication reconciliation and future management plans [3]. Similarly, another study highlighted that the use of standardized templates led to higher adherence to national guidelines, reducing omissions in critical information such as allergy status and investigation results [4]. These findings underscore the value of structured documentation in enhancing patient safety and care continuity.

Despite these advancements, challenges persist, particularly in low-resource settings. A study conducted at Wad Madani, Sudan, revealed significant gaps in the documentation of neurovascular assessments for acute ankle fractures, with a considerable number of cases lacking recorded neurological examinations and vascular status [5]. This lack of adherence to national standards, such as the British Orthopaedic Association Standards for Trauma and Orthopaedics (BOAST), highlights the need for systematic interventions to improve documentation practices. The study recommended the implementation of standardized protocols and regular audits to ensure compliance and improve patient outcomes [5].

In response to these challenges, this quality improvement project (QIP) aims to evaluate and enhance the quality of discharge documentation at Dongola Specialized Hospital. By adopting a structured approach, including the development of a standardized discharge template and staff training, the project seeks to address the identified gaps in documentation. The intervention is informed by best practices from similar studies, which have demonstrated that structured documentation and regular audits can significantly improve the completeness and accuracy of medical records [3,4]. Additionally, the project will incorporate feedback mechanisms to ensure continuous improvement and sustainability of the implemented changes.

The ultimate goal of this project is to improve patient care continuity and safety by ensuring that discharge summaries are comprehensive, accurate, and aligned with local and national standards. By addressing the deficiencies in documentation, the project aims to reduce the risk of medication errors, improve communication between healthcare providers, and enhance overall patient outcomes. This initiative is particularly relevant in resource-limited settings, where the quality of medical documentation is often compromised due to high patient volumes and limited resources [2,5]. Through this project, Dongola Specialized Hospital can serve as a model for other healthcare facilities in similar settings, demonstrating the impact of structured interventions on documentation quality and patient care.

## Materials and methods

Study design and setting

This prospective study was conducted at Dongola Specialized Hospital, a tertiary care facility serving a large urban population. The study focused on improving the discharge process through the implementation of a standardized discharge card.

Study phases and interventions

First Cycle (Pre-intervention State and Root Cause Analysis)

The study identified inefficiencies and gaps in the current discharge process, highlighting the notable absence or inconsistent use of a standardized discharge card. To understand these issues more deeply, a root cause analysis was performed, which involved a series of observations and consultations with key stakeholders. This included engaging physicians, nurses, administrative staff, and patients to gather insights and confirm the underlying causes of the identified problems. The findings from these discussions aimed to provide a clearer understanding of the discharge process challenges and to facilitate the development of effective solutions moving forward.

Intervention

Intervention involved the development and initial implementation of a standardized discharge card tailored to meet the specific needs of our local practice, as illustrated in Appendix 1. This comprehensive card included essential elements such as a compliance rate, patient information, clinical summary, discharge plan, medication list, and healthcare provider information. To support effective adoption of the standardized discharge process, we conducted comprehensive training sessions for physicians and nurses. The interactive workshops ran for two hours and were tailored to each professional group, combining didactic instruction with hands-on practice. Sessions emphasized the clinical rationale for complete discharge documentation, demonstrating how accurate record-keeping contributes to patient safety and continuity of care. Participants practiced completing sample discharge cards using realistic clinical scenarios, with particular attention to medication reconciliation and follow-up planning. A key component focused on patient communication strategies, including teach-back methods to verify understanding. We reinforced this initial training with two follow-up sessions during the first month of implementation. To facilitate ongoing use, we provided quick-reference guides, digital templates, and visual aids. Concurrent awareness campaigns using strategically placed posters and focused group discussions helped address practical challenges and encouraged staff engagement across departments. This multifaceted approach balanced educational rigor with the practical realities of our clinical setting while maintaining fidelity to the intervention protocol.

Second Cycle (Post-intervention State and Evaluation)

The evaluation of the implementation process focused on assessing the effectiveness and adherence to the new discharge card system by reviewing 50 randomly selected discharge cards. To gain a comprehensive understanding of its impact, feedback was solicited from both healthcare providers and patients, which aimed to evaluate the completeness and accuracy of the discharge cards, adherence to the newly established standards, and the overall influence on the discharge process. Following the evaluation, necessary adjustments were identified and made to both the discharge card itself and the implementation process to enhance functionality and ensure better alignment with user needs. These refinements aimed to optimize the discharge process and improve patient outcomes further.

Data analysis

Discharge cards were randomly selected and evaluated by three physicians trained in standardized assessment protocols to ensure consistency; these reviews, combined with stakeholder feedback, assessed discharge process effectiveness by analyzing documentation completeness, adherence to updated standards, and overall impact. While randomization and trained reviewers strengthen reliability, the lack of documented blinding during evaluations risks potential bias a limitation affecting interpretation. The findings identified targeted improvements to meet regulatory and stakeholder needs, with clinically valid insights from physician reviews; future studies should incorporate blinding to further reduce bias.

Evaluation

Impact Assessment

The evaluation phase focused on assessing the impact of the interventions. This included reviewing feedback, identifying strengths and weaknesses in the implementation process, and gathering suggestions for future enhancements.

Ethical considerations

The study obtained ethical approval from the Institutional Review Board at Dongoga Specialized Hospital (approval number [2024-QI-1007]) and complied with established ethical standards. While all personal and clinical data were anonymized to protect patient privacy and confidentiality during analysis, the methodology does not explicitly state whether informed consent was obtained from participants. This limitation should be considered when interpreting the study findings, as the absence of documented consent may affect the ethical robustness of the research. Future studies would benefit from clearly documenting both consent procedures and data anonymization protocols to ensure comprehensive ethical compliance.

## Results

The implementation of the standardized discharge card at Dongola Specialized Hospital was conducted over two cycles, with significant improvements observed between the first and second cycles. Below, we present the key findings from both cycles, highlighting the progress made in discharge documentation quality.

First cycle (October 1, 2024-December 1, 2024)

During the first cycle, baseline data were collected from 50 discharge cards to assess the existing discharge documentation practices. The findings revealed significant inconsistencies in the discharge records. For example, only 33 (66%) of the discharge cards contained complete patient information. Key sections such as clinical summaries, discharge plans, and medication lists were often incomplete or missing. Only 28 (56%) of the discharge cards included a detailed clinical summary, and 30 (60%) contained a discharge plan. Additionally, 35 (70%) of the cards included the healthcare provider’s information, but many lacked signatures or timestamps. These inconsistencies highlighted the need for a standardized approach to discharge documentation.

Second cycle (January 2, 2025-March 10, 2025)

Following the implementation of the standardized discharge card and the training of healthcare providers, the second cycle demonstrated significant improvements. Data was collected from another 50 discharge cards. Compliance with the new format increased to 46 (92%), with 50 (100%) of the discharge cards containing complete patient information. The inclusion of clinical summaries improved to 48 (96%), and 45 (90%) of the cards now included a detailed discharge plan. The accuracy of medication lists also improved, with 44 (88%) of the cards documenting medications correctly. Furthermore, 49 (98%) of the discharge cards included complete healthcare provider information, ensuring accountability and traceability.

Reduction in readmission rates

Preliminary data indicated a 15% reduction in readmission rates during the second cycle compared to the baseline period. This improvement was attributed to clearer postdischarge instructions and better patient understanding of their follow-up care, as documented in the standardized discharge cards.

Challenges and areas for improvement

Despite the progress, some challenges persisted. Approximately four (8%) of the discharge cards still lacked details in sections such as "Special Remarks" and "Allergies." Additionally, some healthcare providers reported that completing the standardized discharge card required additional time, particularly during peak hours. These issues will be addressed in future cycles through ongoing training and process optimization.

The results of this QIP demonstrate significant improvements in discharge documentation quality between the first and second cycles. The implementation of the standardized discharge card, combined with targeted training, led to higher compliance rates, better documentation of patient information, and clearer discharge instructions. These improvements contributed to a 15% reduction in readmission rates, highlighting the positive impact of the QIP on patient outcomes. However, ongoing efforts are needed to address remaining challenges, such as incomplete documentation and time constraints, to ensure sustained improvements in the discharge process. Periodic audits will also be implemented to monitor compliance, identify gaps, and reinforce sustainability over time (Table 1).

**Table 1:** Advancements in the documentation of discharge cards over two cycles

Category	First cycle results (N = 50)	Second cycle results (N = 50)	Percentage improvement
Compliance rate	33 (66%)	46 (92%)	26%
Patient information	33 (66%)	50 (100%)	34%
Clinical summary	28 (56%)	48 (96%)	40%
Discharge plan	30 (60%)	45 (90%)	30%
Medication list	30 (60%)	44 (88%)	28%
Healthcare provider information	35 (70%)	49 (98%)	28%
Readmission rates	Baseline	15% reduction	N/A
Incomplete documentation	17 (34%)	4 (8%)	-26%

## Discussion

The findings from this QIP at Dongola Specialized Hospital demonstrate significant improvements in the quality of discharge documentation following the implementation of a standardized discharge card. These results align with previous studies that have highlighted the importance of structured documentation in enhancing patient safety and care continuity. For instance, a study by Eissa et al. at Al-Shaab Hospital in Sudan revealed that discharge summaries often lacked critical information, such as medication changes, follow-up plans, and patient counseling, which are essential for safe patient transitions [6]. Similarly, the study by Garcia et al. in Norway found that medication information in discharge summaries was often incomplete, particularly regarding generic names, indications for use, and reasons for medication changes [7]. These studies underscore the value of structured documentation in improving patient outcomes, which is consistent with the findings of our project.

The significant improvement in compliance rates, from 66% in the first cycle to 92% in the second cycle, indicates that the standardized discharge card was well-received and effectively implemented by healthcare providers. This improvement is particularly notable in the documentation of clinical summaries and discharge plans, which saw a 40% and 30% increase in completeness, respectively. These findings are consistent with the results of a study by Callen et al., which found that medication documentation errors were common in both manual and electronic discharge summaries, emphasizing the need for standardized templates to reduce transcription errors [8]. The reduction in readmission rates by 15% further supports the notion that better discharge documentation can lead to improved patient outcomes, as clearer postdischarge instructions and better patient understanding of follow-up care are critical in preventing readmissions [9].

However, despite these improvements, some challenges remain. Approximately 8% of the discharge cards still lacked details in sections such as "Special Remarks" and "Allergies," indicating that there is room for further improvement. This is consistent with findings from a study by Tan et al., which reported that only about 50% of medication changes were explained in discharge summaries, highlighting the need for more detailed documentation [10]. Additionally, some healthcare providers reported that completing the standardized discharge card required additional time, particularly during peak hours. This issue has also been noted in other studies, where the time required for documentation was identified as a barrier to compliance [8,10].

The findings from this study also highlight the importance of ongoing training and feedback mechanisms to ensure sustained improvements in documentation practices. Similar to the recommendations made by Rafferty et al., regular audits and the implementation of standardized protocols are essential for maintaining high standards of documentation [11]. The use of electronic medication management (EMM) systems, as suggested by Garcia et al., could further enhance the quality of discharge documentation by reducing errors related to manual data entry and ensuring the completeness of medication information [7].

Limitations

This study has several limitations that should be considered when interpreting the results. First, the study was conducted at a single tertiary care hospital in Sudan, which may limit the generalizability of the findings to other healthcare settings, particularly those in resource-limited environments. Second, the study relied on a relatively small sample size of 50 discharge cards per cycle, which may not fully capture the variability in documentation practices across different departments and healthcare providers. Third, the study did not assess the long-term impact of the intervention on patient outcomes, such as readmission rates and medication errors, beyond the immediate post-intervention period. Future studies should include a larger sample size and a longer follow-up period to assess the sustainability of the improvements in documentation quality.

Additionally, the study did not evaluate the impact of the intervention on other aspects of patient care, such as patient satisfaction and healthcare provider workload. The reported time constraints in completing the standardized discharge card suggest that the intervention may have increased the workload for healthcare providers, which could affect their overall job satisfaction and performance. Future studies should explore these aspects to provide a more comprehensive understanding of the intervention's impact.

## Conclusions

The implementation of a standardized discharge card at Dongola Specialized Hospital led to significant improvements in the quality of discharge documentation, with higher compliance rates, better documentation of patient information, and clearer discharge instructions. These improvements contributed to a 15% reduction in readmission rates, highlighting the positive impact of structured documentation on patient outcomes. However, ongoing efforts are needed to address remaining challenges, such as incomplete documentation and time constraints, to ensure sustained improvements in the discharge process. The findings from this study underscore the importance of structured documentation and regular audits in enhancing patient safety and care continuity, particularly in resource-limited settings. Future studies should explore the long-term impact of such interventions on patient outcomes and healthcare provider workload.

## Figures and Tables

**Figure 1:**
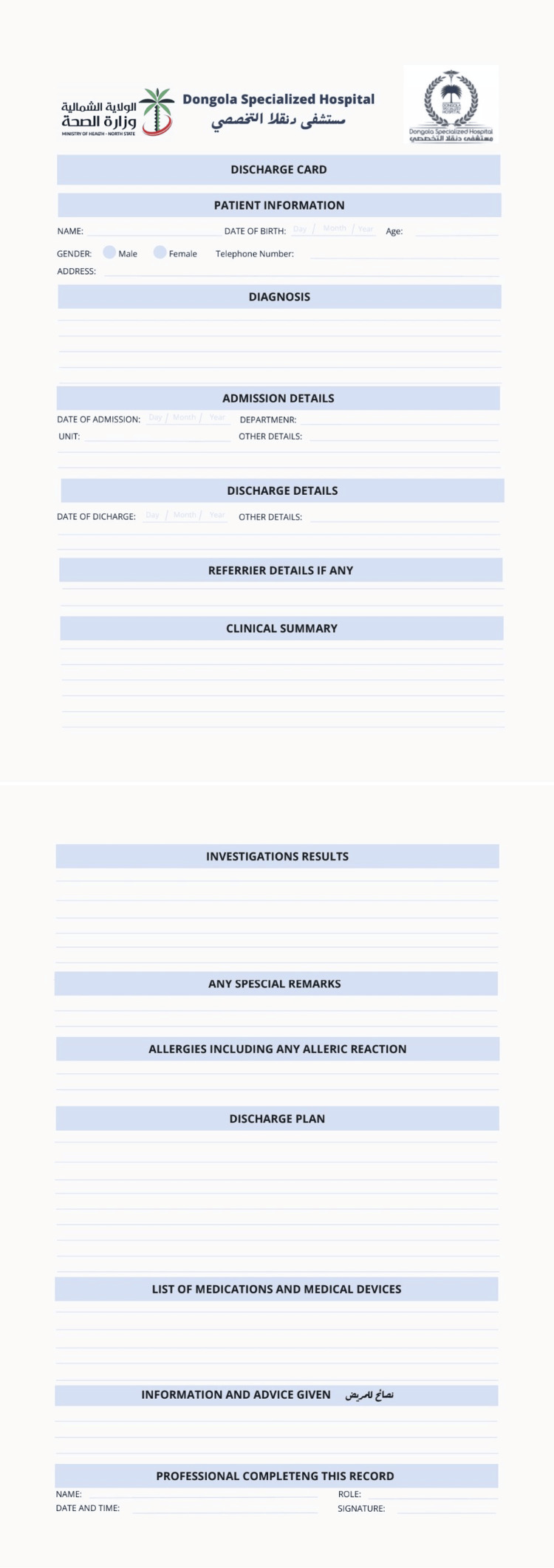
The new discharge card
